# 5-year survival rates based on the type of leukemia in Iran, a Meta-analysis

**DOI:** 10.22088/cjim.9.4.316

**Published:** 2018

**Authors:** Yousef Veisani, Salman Khazaei, Ali Delpisheh

**Affiliations:** 1 Psychosocial Injuries Research Center, Ilam University of Medical Sciences, Ilam, Iran; 2 Department of Epidemiology, School of Public Health, Hamedan University of Medical Sciences, Hamedan, Iran; 3 Department of Clinical Epidemiology, Ilam University of Medical Sciences, Ilam, Iran

**Keywords:** ALL, AML, Leukemia, Survival, Iran

## Abstract

**Background:**

According to epidemiological studies, leukemia is among the five most common cancers in Iran. Keeping efforts to estimate survival is critical to monitoring and improving patients’ quality of life with leukemia. The purpose of this study was to evaluate the 5-year survival rate of leukemia patients in Iran using meta-analysis method.

**Methods:**

This meta-analysis was carried out according to studies that adhere to inclusion and exclusion criteria during enrolment. The valid Iranian databases included: Medex, Magiran, SID, and Medlib, along with international data bases, namely, Scopus, Pubmed, and ISI were searched to find relevant articles. After determining the heterogeneity between studies, the random effects models were used to estimate pooled survival in leukemia patients.

**Results:**

In total, 18 studies involving 2517 participants were included in this meta-analysis. The pooled 5-year survival rate was 0.56 % (95% CI, 0.54 to 0.58). According to types AML and ALL, the 5-year survival rates in Iran were 35.0 % (95% CI: 32.0-38.0) and 57.0 % (95% CI: 54.0-60.0), respectively.

**Conclusion:**

Based on our findings, slightly less than 50% of leukemia deaths happened in the first 5 years after diagnosis, which is lower than the global average.

Acording to National Cancer Registry reports; stomach, esophagus, colon-rectum, bladder and leukemia cancers are the five most common types of cancer in Iranian males (1). Distribution of cancers in the different provinces of Iran does not have the same pattern, i.e in Fars province, leukemia ranks the first and second cancer in males and females, respectively (2). Generally, this malignancy is more common among the developed countries (3). The results of age-adjusted death rate, based on 2009-2013 USA statistics of deaths, showed that the incidence rate of leukemia was 13.5 per 100,000 in men, and also 6.9 per 100,000 in both genders (4). In recent decades, the 5-year survival rate of leukemia patients has increased due to the improvement of medical procedures and treatments. So the 5-year survival rate in individuals with age less than 20 years has reached to 89% (5, 6). Supportive cares in the USA have improved the 5-year survival rate in patients with leukemia up to 70% in recent years (7). In Iran, a 5-year survival rate is lower than the developed countries, hence, according to reports, the index rates for ALL and AML patients were 53.3% and 25.0%, respectively (8, 9). The important reason for this increasing rate of survival in leukemia patients wase associated to rigorous treatment accompanied stem cell transplantation (10).

The important factors that are acknowledged can be predicting survival in leukemia patients including; some patients’ resistance to treatment in laboratory factors (number of white and red blood cells, the means of corpuscular hemoglobin, sodium, potassium and calcium); smoking, patients’ age, bone marrow transplants and history of disease (11). However, the exact effect of some of these factors on survival is unclear; sex and gender (12), may be due to different methods in different studies.

Keeping the researcher's effort to estimate survival is very useful in monitoring and improving the quality of life of patients with leukemia, which can lead to conducting better screening programs and discovering new treatments. In this meta-analysis, we evaluated recent relevant studies towards the 5-year survival rate of leukemia in Iran.

## Methods

**Data sources: **This meta-analysis was carried out to estimate the survival rates of leukemia in Iran according to studies that adhered to inclusion and exclusion during enrolment according to the preferred reporting items for systematic reviews and meta-analyses (PRISMA) guidelines (13). The inner covering; Iran Medex, Magiran, SID, and Medlib, also international data bases including; Scopus, Pubmed, and ISI were searched to find the relevant articles.

**Search strategy: **Title searching for studies was done by MeSH heading leukemia and other keywords including; Iran, survival rate, and rate. The search strategies in pubmed were ("survival rate"[MeSH Terms] OR ("survival"[All Fields] AND "rate"[All Fields]) OR "survival rate"[All Fields]) AND ("leukaemia"[All Fields] OR "leukemia"[MeSH Terms] OR "leukemia"[All Fields]) AND ("Iran"[MeSH Terms] OR "Iran"[All Fields]). All titles and next abstracts were checked to find out the most relevant articles. Next, the full texts of related articles were assessed including relevant articles in meta-analysis. In the final step, cross-referring was done to increase search sensitivity.

**Inclusion and exclusion criteria: **At the first step to enhance sensitivity of search, all epidemiologic studies regarding survival for leukemia patients in Iran in Persian and English language were enrolled. The time period of this study was 2008-2016. After primary search, we reviewed the full text and the inclusion criteria were; at least a 5-year follow-up of patients, estimation of 5-year survival rate. In this study, studies with less than 5-years of follow-up period, and also articles of poor quality (based on NOS scale) were excluded.

**Data extraction: **Screening and review of studies was done by two authors independently. The key information about the first author’s name, year of publication, study design, patients characteristics, period of data acquisition, and 5-year survival were extracted in a standardized form. Afterwards, the details were imported to the software to apply meta-analysis.

**Quality assessment: **The methodological methods included studies assessed by Newcastle-Ottawa Scale(14). Eventually the articles were classified to three groups of studies, high (>7 points), medium (6-7 points), and low quality (<6 points). 

**Statistical analysis: **The heterogeneity between studies was calculated by I² statistics. Since the heterogeneity in pooled estimate and in subgroups was higher than 25 percent, therefore the random effects model was applied to estimates of the overall 5-year survival rate (15). The Begg’s test was used to assessing publication bias in this study (16). Analysis was done by Stata software Version 11.2 at the significance level of 5%.

## Results

**Figure 1:**
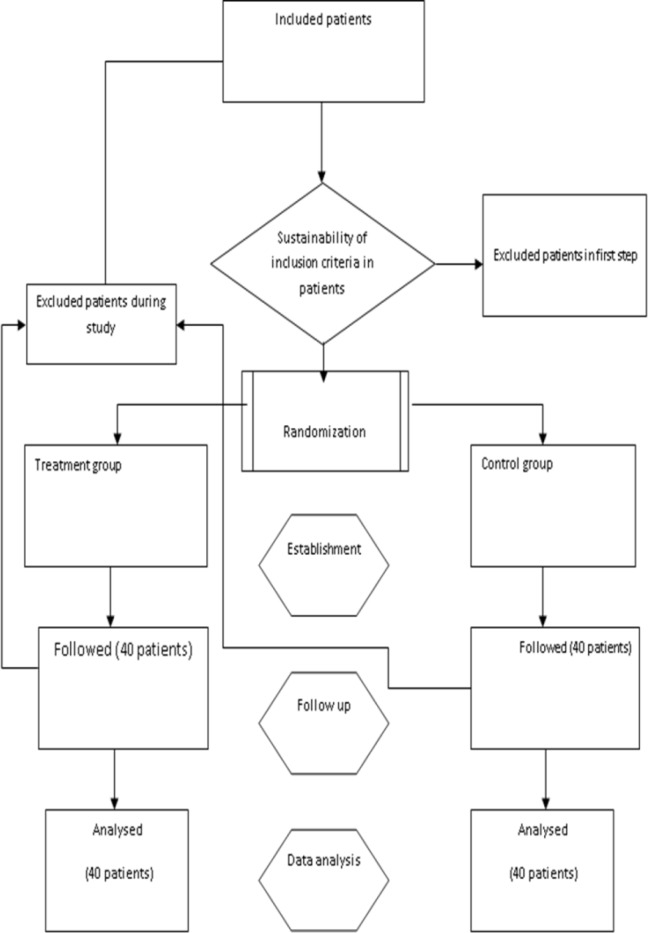
follow chart of included studies

**Figure 2:**
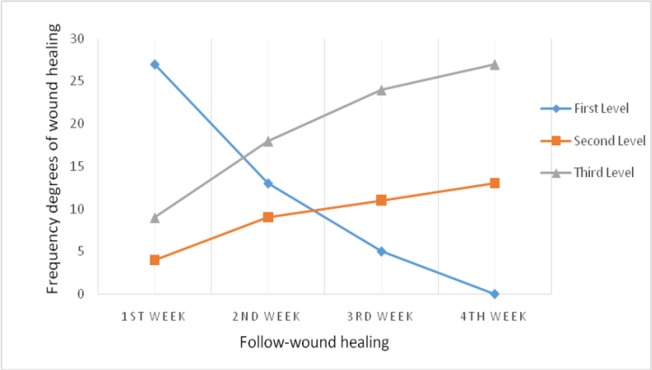
5-year survival rates of leukemia patients in Iran

In the initial search, 290 titles in all databases were recognized, they were screened to enrol more relevant studies and 103 abstracts were recognized and added as relevant studies according to meta-analysis. Abstracts were reviewed to find the best studies that met our inclusion criteria. Full texts of 37 articles were reviewed to identify final meta-analysis studies. We excluded 19 articles because of their irrelevance according to exclusion criteria. After excluding the studies that did not fulfill the inclusion criteria, finally 18 relevant studies were determined for meta-analysis (figure 1). Important characteristics of included studies were shown in table 1. The study duration was from 2008 to June 2016. Of total 2517 survivors, 836 were ALL, 1070 were AML, 190 were CLL and about 502 were CML. Of the 18 included studies, seventeen articles had a cross-sectional design and one of them was retrospective design. Stage of flow up in three studies was different, one study was hematopoietic stem cell transplant (HSCT) (17), another one was after chemotropic treatment program (18) and the third study was after treatment with imatinib mesylate (19). In other 15 studies, the patients were identified during the hospital-initiated treatment. Data in all studies were based on hospital and medical record. Age range in 8 articles was below 15 years old while more than 15 years old in 10 studies. The overall 5- year survival rate in leukemia patients in Iran was 0.56% (0.54 to 0.58, 18 studies). The heterogeneity between studies was significant (X^2^ = 64.37, p<0.001, I ^2^ = 97.7%, 95% CI 96.4–98.3) (figure 2). 

**Figure 3:**
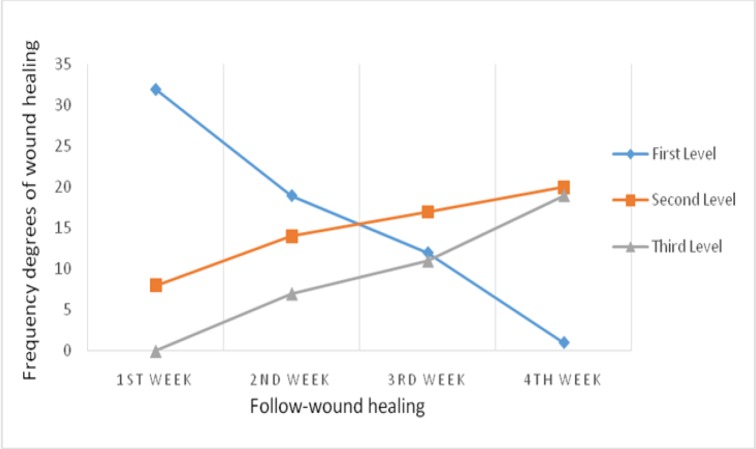
5-year survival rates of leukemia patients in Iran according to type

5-year survival rates based on type of leukemia are shown in figure 3. Based on the AML and CML the 5-year survival rate in Iran were 35.0 % (95% CI: 32.0-38.0, 9 studies) and 83.0 % (95% CI: 79.0-86.0, 2 studies), respectively. 5-year survival rates for other types included ALL and CLL were 57.0 % (95% CI: 54.0-60.0, 6 studies) and 64.0 % (95% CI: 55.0-73.0, 1 studies) respectively.

Possibility of sources for heterogeneity was assessed by subgroup analysis. When we obtained the heterogeneity with regard to quality of papers, the positive heterogeneity did not show yet (p<0.001). According to **table 2**, a 5- year survival rate paper with high quality is lower than the articles with low quality and also higher than medium quality papers. In patients aged below or more than 15 years old, 5- survival rate were 61.0 % (95% CI: 58.0-64.0, 8 studies) and 53.0 % (95% CI: 51.0-55.0, 10 studies), respectively.

**Figure 4:**
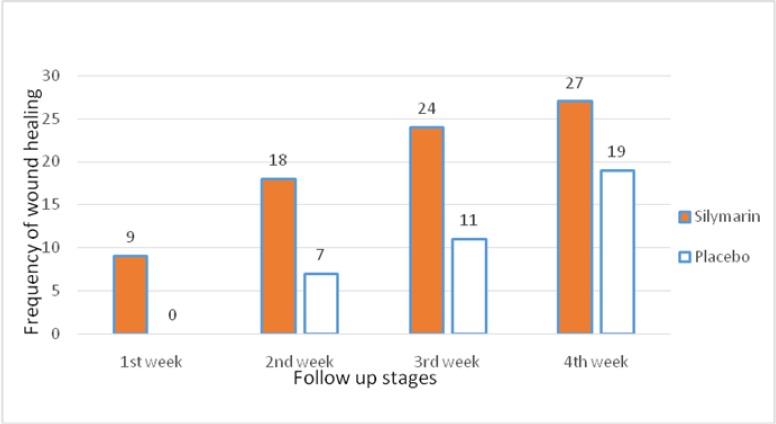
Results of meta-regression by plots, the covariates are subjects and year of publication

Results of meta-regression are shown in figure 4 according to the year of publication and the number of subjects did not have association with heterogeneity in outcome. Thus, the year of publication and number of subjects were not related to causes of variability in the five year survival rate results (reg coef = 0.347, p=0.65) and (Reg Coef = 0.358, p=0.128), respectively (figure 4). 

**Figure 5:**
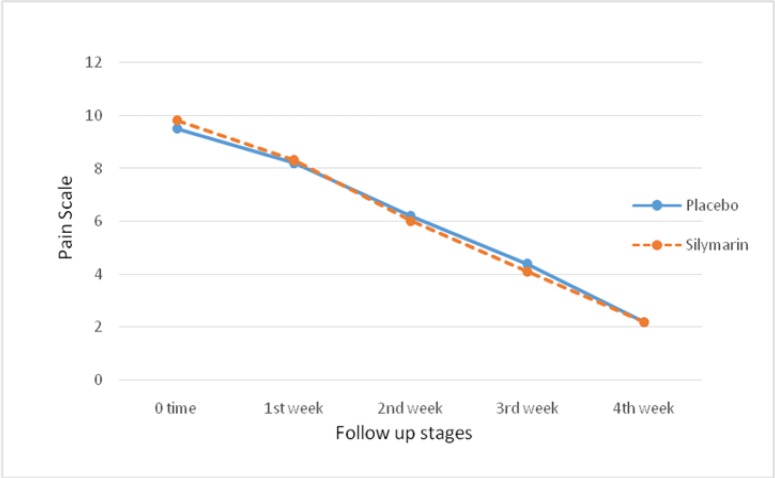
Funnel plot according to Begg's approach showing the mean differences in the 5-year survival rates according to enrolled articles

Publication bias was assessed by funnel plots, according to them we did not find evidence for publication bias (bias: 3.21, 95% CI= -09.14–18.12; p=0.668), consequently, our research study was considered the most published article in this subject (figure 5).

**Table 1:** Characteristics of studies included in meta analysis

**Ref.**	**Author name,** **Year of Pub**	**Leukemia type (N)**				**Years of flow sitting**	**Age**	**5-year survival rate**	**Quality of final articles**	**Stage of evaluation**
		**ALL**	**AML**	**CLL**	**CML**					
(20)	Payandeh, M. 2015			109		2006-2014 Kermanshah	15<	64	Medium	After treatment in hospitalized patients
(8)	Hashiani, A. 2013	179	64			2004-2008 Shiraz	15>	53.3	High	After treatment in hospitalized patients
(21)	Allahyari, A. 2016,		96			2009-2015 Mashhad	15<	26.6	Medium	After treatment in hospitalized patients
(22)	Karimi, M. 2012	76				1995-2000 Shiraz	15>	72.5	High	After treatment in hospitalized patients
(23)	Payandeh, M. 2015,				85	2002-2014 Kermanshah	15<	79.7	Medium	After treatment in hospitalized patients
(24)	Akramipour,R, 2007		40			1996-2000 Ahvaz	15>	35	Low	After treatment in hospitalized patients
(25)	Sanaat, Z. 2010		207			2001-2009 Tabriz	15<	24.6	Low	After treatment in hospitalized patients
(26)	Saffar, A. 2015		85			2008-2013 Tehran	15<	22	Medium	After treatment in hospitalized patients
(27)	Hashemi AS, 2009		56			2001-2007 Yazd	15>	88.5	Low	After treatment in hospitalized patients
(17)	Sayehmiri, K. 2008	206				1993-2007 Tehran	15<	52	High	after hematopoietic stem cell transplant (HSCT)
(18)	Ashrafi, F. 2013		94			2002-2010 Isfahan	15<	18	High	After chemotropic treatment
(28)	Teshnizi,S. 2013	102	95			2006-2009 Isfahan	15>	50.3	Low	After treatment in hospitalized patients
(29)	Mashhadi, M.A. 2012		66			2003-2010 Zahedan	15<	44.8	High	After treatment in hospitalized patients
(9)	Sanaat, Z. 2011		142			2001-2009 Tabriz	15<	25	Low	After treatment in hospitalized patients
(30)	Mousavinasab, N. 2015	84	13			2006-2014	15>	79.7	Low	After treatment in hospitalized patients
(31)	Ansari,Sh, 2007		83			1988-2003 Tehran	15>	58	Medium	After treatment in hospitalized patients
(19)	Jalaeikhoo, H. 2011				417	2004-2010 Tehran	15<	83	High	After treatment with imatinib mesylate
(32)	Parvareh,M. 2015,	189	29			1998-2009 Kerman	15>	51	High	After treatment in hospitalized patients

## Discussion

The present meta-analysis demonstrated the 5-year survival rates of leukemia in Iran. The pooled 5-year survival rate in patients with leukemia in Iran was 56%. Previous reports in other parts of the world have reported that the 5-year survival rates of leukemia in Turkey were 36.5% (33), China 81.8% (34), India 51% (35) and 69.9% in Korea (3). We found heterogeneity in included studies. The important sources of heterogeneity in leukemia survival by meta-regression in primary articles were assessed and according to meta-regression findings; publication year, quality of articles, and number of patients in each study were not important sources of heterogeneity. The other potential sources of heterogeneity in leukemia can be sources of data, length of follow-up and also stage of cancer diagnosis which we could not assess due to lack of data in individual studies. Subgroup analysis showed that five–year survival rates based on AML and ALL in Iran was 35.0% and 83.0%, respectively, that ALL patients have shown a better prognosis. Moreover, based on patients age, the overall survival rates for patients below and more than 15 years old five were 61.0% and 53.0%, respectively, indicating those patients below 15 years old is associated with better patient survival rate. One report, based on large groups of AML patients in the USA, showed that 65% of those aged less than 15 years survive from leukemia after 5 years, and this proportion for patients in 15-25 year’s age group, 25-65 years age group, and up to 65 years were 60%, 40% and 5%, respectively. ALL has better outlook and five survival rates were 70% after diagnosis overall. This proportion for 15 years, or younger than 15 years old was 90%, 15-25 years age group was 70%, 26-64 years age group was 40%, and in patients aged 65 or older was 15% after diagnosis (4).

As shown previously, survival in younger individuals is better than older patients (36), genetic abnormalities diminish survival in leukemia patients, late diagnosis have a bad outlook for survival in all types of cancers (37, 38), plus high count of white blood cells is a predictor for better survival (39). The important limitations in current study are at first, some age groups (5<) were excluded due to the limitation in years of follow-up. Second, we evaluatd long term survival and survival less than 5 year was not estimated in the present meta-analysis. Third, some of the important predictors for heterogeneity such as data sources, length of follow-up and stage of cancer diagnosis were not assessed due to the lack of enrolled studies. Fourth, data sources in most primary studies were based on hospital records, as a result, they have some difficulties in their generalizability because data were not gathered for the purpose of investigation. Finally, typing mistakes and missing data are the other limitations in these sources.

We can conclude that we enrolled 18 studies so that we could get reliable results about the 5-year survival rates of leukemia in Iran. We found that a 5-year survival rate in AML patients is lower than ALL patients, as well as 15 and below of age have shown a better prognosis compared with adults. According to these results, future research should be conducted to increase survival in leukemia patients; furthermore, guidance for clinicians can help improve long-term survival in patients with leukemia.

## Conflict of Interest:

None.
